# Maxillary implant-retained overdentures: telescopic versus LOCATOR attachments

**DOI:** 10.1038/s41432-026-01228-w

**Published:** 2026-06-10

**Authors:** Nidhi Parmar

**Affiliations:** MClinDent Prosthodontics Specialty Trainee, Eastman Dental Institute, London, UK

**Keywords:** Removable prosthodontics, Dental implants, Dental implants, Removable prosthodontics, Peri-implantitis

## Abstract

**A Commentary on:**

**Ibrahim WI, Abdeen RM, Denewar MM, Kortam SA**.

Retention of telescopic versus LOCATOR attachments and marginal bone loss surrounding implant-retained maxillary overdentures: A 2-year randomized clinical trial. *J Prosthet Dent*. 2026;135:778–786.

**Design:**

Prospective two-arm parallel randomised controlled trial (RCT) comparing telescopic and LOCATOR attachments for four-implant retained maxillary overdentures over 24 months.

**Case selection:**

40 patients with complete edentulism received four maxillary implants to retain a maxillary overdenture and two mandibular implants to retain an opposing mandibular overdenture. Patients were randomly allocated to telescopic or LOCATOR maxillary attachment groups. Inclusion criteria included adequate maxillary bone volume and quality, suitable inter-arch space, acceptable systemic health and absence of parafunctional habits or conditions affecting osseointegration.

**Data analysis:**

Retention was measured using a digital force gauge at R0, one month after prosthesis delivery, and at 12 and 24 months. Vertical and horizontal marginal bone loss were assessed using standardised periapical radiographs and digital software. Between- and within-group comparisons were performed using t tests with Bonferroni correction.

**Results:**

Telescopic attachments showed significantly greater retention than LOCATOR attachments at all time points. Telescopic retention decreased from 28.59 N to 15.63 N over 24 months, while LOCATOR retention increased slightly at 12 months before decreasing to 9.44 N. Marginal bone loss was significantly greater in the telescopic group at 12 and 24 months. One implant failed in the telescopic group.

**Conclusions:**

Telescopic attachments provided greater retention, whereas LOCATOR attachments were associated with less marginal bone loss. Both systems remained within accepted implant success thresholds over two years.

## GRADE Rating:



## Commentary

Maxillary implant-retained overdentures are less predictable than mandibular implant-retained overdentures. The mandibular two-implant overdenture is supported by strong evidence and broad consensus, whereas guidance for the edentulous maxilla remains less definitive^1^. This reflects differences in bone quality, prosthesis support and load distribution. The maxilla has poorer trabecular bone, greater prosthesis movement, less favourable anterior-posterior implant spread and greater susceptibility to non-axial loading^2,3^. Attachment selection therefore affects not only retention, but also prosthesis movement, load transfer and peri-implant strain.

Ibrahim et al.^4^ evaluated whether telescopic and LOCATOR attachments differ in retention and marginal bone loss for four-implant retained maxillary overdentures. Telescopic attachments provided greater retention but were associated with greater marginal bone loss over two years, challenging the assumption that higher retention is necessarily the better prosthodontic outcome. This question is important because evidence for maxillary implant-retained overdenture attachments remains limited, with much of the literature drawn from retrospective studies, laboratory data or mandibular implant-retained overdenture research^5,6^. Direct extrapolation is problematic because maxillary overdentures are more vulnerable to prosthesis rotation, increased rotational leverage and non-axial implant loading^7^. Outcomes are also influenced by implant number, prosthesis design and maintenance factors, not attachment choice alone^8^. This trial therefore, addresses a relevant clinical gap.

The protocol was carefully standardised and has several methodological strengths. Randomisation, clinical relevance and procedural standardisation support a medium quality rating. Both groups received four maxillary implants, comparable implant positioning, the same implant system, delayed loading and an opposing mandibular overdenture; guided surgery further reduced procedural variation. These features strengthen internal validity, but the modest sample size, 24-month follow-up, narrow outcome set and reliance on radiographic surrogate endpoints limit generalisability. The findings therefore, apply most directly to favourable cases with four parallel implants and complete palatal coverage.

The numerical findings need careful clinical interpretation. The sample size was calculated to detect differences in retention, not implant survival, prosthetic complications or clinically meaningful thresholds of marginal bone loss. Although bone loss differences were statistically significant, their absolute magnitude was modest. At 24 months, vertical bone loss differed by approximately 0.44 mm and horizontal bone loss by approximately 0.24 mm. Although statistically significant, a sub-millimetre radiographic difference alone does not establish a change in prognosis, maintenance burden or treatment choice.

The study appropriately addressed its stated aim by measuring retention and marginal bone loss. However, these outcomes alone do not define prosthodontic success. Maintenance, hygiene access, prosthetic complications, patient adaptation and long-term peri-implant stability all influence overdenture success^2,5^. These outcomes were outside the scope of this RCT, but their absence limits any claim of overall attachment superiority.

The greater retention of telescopic attachments is likely explained by frictional contact and wedging between the primary and secondary copings. The key finding is that this higher retention was associated with greater marginal bone loss. This is biomechanically relevant because telescopic attachments create a more constrained friction-retained coupling, limiting rotational freedom during function. Conversely, LOCATOR attachments incorporate resilient nylon inserts and a low-profile stud design, allowing limited vertical and rotational movement between the denture base and implant abutments. In the maxilla, where prosthesis displacement and non-axial loading are common, this difference in attachment behaviour may influence how functional loads are transferred to peri-implant bone. Existing biomechanical evidence supports this interpretation. Studies of maxillary implant-retained overdentures show that attachment design, palatal coverage and implant distribution influence implant strain^9–11^. Ying et al. also reported that attachment height and geometry affect lateral force transmission around overdenture implants^12^. These findings support the principle that, in the maxilla, controlled prosthesis movement may reduce strain concentration more effectively than simply increasing resistance to displacement.

However, the findings should not be read as proof that LOCATOR attachments are superior. LOCATOR systems require maintenance. Nylon inserts lose retention with wear, cleansing exposure and implant divergence^13,14^. Telescopic attachments remain useful in selected patients, especially where high retention, a defined path of insertion and improved resistance to functional displacement are priorities. Notably, telescopic retention decreased from 28.59 N to 15.63 N over 24 months, an almost 45% reduction. This suggests that friction-retained systems may retain an early mechanical advantage while still showing meaningful wear over time.

Several design features limit direct translation to all clinical cases. Retention was measured using a central vertical pull-off test, which is reproducible but does not replicate function. Maxillary overdentures are displaced by vertical, oblique, horizontal and rotational forces during mastication and functional mandibular movement. Therefore, a single vertical dislodgement value cannot fully describe clinical stability. Complete palatal coverage is also relevant, as it likely maintained some implant-mucosal support and may have reduced implant loading in both groups. Results may differ in palateless designs, where support becomes more implant-dependent and lateral load transfer may increase^9–11^. Similarly, the use of parallel implants in favourable positions may underrepresent the practical advantages of resilient stud attachments in cases with implant divergence or rotational discrepancy^14^.

The marginal bone loss data should be interpreted carefully. The authors noted that bone loss remained within accepted thresholds for implant success. Historically, peri-implant bone loss of 1–1.5 mm during the first year, followed by annual loss below 0.2 mm, has been used as a success criterion^15^. These thresholds are useful but incomplete. Marginal bone behaviour is influenced by loading, prosthetic design, inflammation, maintenance and patient risk factors^16^. The additional bone loss around telescopic attachments may reflect greater crestal stress concentration, but its long-term significance cannot be determined from a two-year study.

The value of this paper lies in reframing attachment choice in maxillary implant-retained overdentures. It does not identify a universally superior attachment. It shows that greater retention may carry a biological cost. In the maxilla, successful overdenture design should not aim simply for maximal retention. It should aim for favourable force distribution, maintainability and controlled prosthesis movement^2,5,9–12^.

Practice point

- In maxillary implant-retained overdentures, attachment selection should not be based on retention alone. This study suggests that although telescopic attachments may provide greater retention, LOCATOR attachments may offer a biological advantage by permitting controlled prosthesis movement and showing less marginal bone loss.
